# Participation and Activity Inventory for Children and Youth (PAI-CY): Translation and Cultural Adaptation to European Portuguese

**DOI:** 10.3390/brainsci15040394

**Published:** 2025-04-13

**Authors:** Ana Isabel Ferreira, Maria Raquel Santana, Cláudia Quaresma, Carla Quintão

**Affiliations:** 1 Physics Department, NOVA School of Science & Technology, NOVA University of Lisbon, 1099-085 Lisbon, Portugalcmquintao@fct.unl.pt (C.Q.); 2 LIBPhys, LA-REAL, NOVA School of Science & Technology, NOVA University of Lisbon, 1099-085 Lisbon, Portugal; 3 Health School, Polytechnic Institute of Beja, 7800-295 Beja, Portugal; 4 Quality of Life Research Centre, Polytechnic Institute of Beja, 7800-295 Beja, Portugal; 5 Interdisciplinary Centre for Social Sciences, NOVA University of Lisbon, 1099-085 Lisbon, Portugal

**Keywords:** participation and activity inventory for children and youth (PAI-CY), visual impairment, assessment, translation, cultural adaptation

## Abstract

**Introduction:** Visual skills have a significant impact on an infant’s development and participation. In Portugal, although the public health system is organized to support citizens with visual impairment, it lacks translated and culturally adapted assessment tools. Knowing that a comprehensive assessment is crucial for early diagnosis and that it improves an infant’s participation in daily life, the aim of the present article is to present the translation and cultural adaptation of the Participation and Activity Inventory for Children and Youth (0–2 years) into European Portuguese. **Methods:** To achieve the objective, methodological guidelines were followed, and six different stages were implemented, including translation, synthesis, back translation, revision by an expert committee, pretesting in a representative sample, and submission and appraisal of all written reports by developers to the author’s tool. The whole process was previously approved by an Ethics Committee. **Results:** A Portuguese version of the Participation and Activity Inventory for Children and Youth (0–2 years) was obtained based on the English version of PAI-CY (0–2 years) with the participation of four independent translators, five experts, and twenty parents filling out the pretest version. The results of the expert committee showed that 99.40% were in favor of the proposed translation. Additionally, the inventory pretesting reveals a clarity PAI-CY validity index of 1, a relevance PAI-CY validity index of 0.98, and a global validity index of 0.99. **Conclusions:** The Participation and Activity Inventory for Children and Youth (0–2 years) has now been translated and culturally adapted into European Portuguese, guaranteeing its content validity. To use the tool in Portugal with certainty it is necessary to implement the tool validation. The process to investigate the psychometric properties of the questionnaire is being prepared.

## 1. Introduction

Visual skills are crucial for daily life performance and childhood development [1,2]. The link between visual ability and the development of children in terms of their motor, cognitive, and learning abilities has been well established [3,4]. However, in developed countries visual impairment is increasing among infants, mainly due to cerebral impairments [5]. Most of the time, this results from dysfunctions caused by preterm birth, hypoxia or anoxia during delivery, or genetic disorders [6,7,8,9]. It has lifelong and far-reaching implications, for both children and their parents [10].

Independent of the reason, early diagnosis and attempted therapeutic intervention is crucial to improve performance and, when possible, recovery [11].

According to the rehab cycle proposed by the World Health Organization (WHO), assessment is a crucial step in any therapeutic approach [12]. To implement a complete and holistic assessment, different kinds of methods and procedures can be used in order to collect information about the child from a holistic perspective. In a previous systematic review regarding visuomotor and visual perception skills assessment in children up to 6 years old, tests such as the Beery–Buktenica Developmental Test of Visuo-Motor Integration and the Developmental Test of Visual Perception were the most used [13]. Additionally, the use of questionnaires, such as the Preverbal Visual Assessment, was also reported. Caregivers’ questionnaires allow relevant information about the infant’s daily life to be collected, which can offer a deeper knowledge of child performance and optimize the therapeutic assessment. The use of questionnaires as one of the tools for data collection was recommended [14,15]. In a seminal study, participants from the American Academy of Pediatrics were recruited, and approximately 40% of practitioners confirmed that they used questionnaires to collect clinical information about patients, highlighting the efficiency of this kind of tool [16]. Similarly, recent research on parental involvement in the rehabilitation process demonstrated that parental engagement correlates with improved outcomes and enhances the relevance of parental reports [17]. The use of questionnaires or inventories completed by caregivers and other adults serves as a valuable method for gathering information, aiding in the identification of presenting problems, estimating their duration, and clarifying family priorities [18].

Despite the recognition of the value of questionnaires in the assessment process, there are few instruments available in European Portuguese. As far as we know, none of them are specific to the assessment of visual abilities.

In recent years, several questionnaires have been specifically developed to gather information about the symptoms of visual impairment and its impact on daily life. Some of these tools include the Preverbal Visual Assessment, the Children’s Visual Function Questionnaire, the Pediatric Eye Questionnaire, and the Children’s Visual Impairment–Vision Function Questionnaire. Among the various options currently available, the Participation and Activity Inventory for Children and Youth (PAI-CY), developed in the Netherlands, stands out for its specific focus on the structural domains of the activity and participation chapter of the International Classification of Functioning, Disability, and Health [19]. PAI questionnaires were developed to assess the needs of visually impaired children and youths aged 0–17 years (PAI-CY), and young adults aged 18–25 years (PAI-YA). To aid interpretation of the PAI-CY, four different age categories were formed based on the WHO criteria: 0–2 years (infants and toddlers), 3–6 years (preschool children), 7–12 years (school-aged children), and 13–17 years (adolescents). For each age category, a specific version was developed, which differs in the domains covered and the number of items included [19]. The present work focuses on PAI-CY 0–2 years because the research team believes it is important to follow the typical development path and thus start the process with the tool designed for younger children. This age-specific version allows for the structured collection of information regarding the potential impact of visual impairment on infant participation in various occupational areas and across different life contexts.

As is known, the use of assessment tools follows specific rules in order to guarantee both content validity and reliability. One of the rules is to administer the instrument in the native language of the user. To do that, a scientific process of translation, transcultural adaptation, and validation is required, and different approaches could be used. To conduct the translation and cultural adaptation, a guideline composed of six stages, which are translation, synthesis, back translation, expert committee review, pretesting, and submission and appraisal of all written reports by the developer’s committee, was followed [20].

The objective of this study was to translate the English PAI-CY questionnaires for children aged 0–2 years, to adapt items to make them relevant to the Portuguese context, and pretest the questionnaires to determine its feasibility.

## 2. Materials and Methods

The authorization to perform the translation, cultural adaptation, and validation was given by the authors in October 2020. After that, the Portuguese research team submitted the project to the Ethics Commission of the Polytechnic Institute of Beja, and authorization was obtained.

### 2.1. Participation and Activity Inventory for Children and Youth (0–2 Years)

The present translation and cultural adaptation were focused on the 0–2 years Participation and Activity Inventory for Children and Youth (PAI-CY)–English version, which contains 27 items categorized into seven domains: stimulus processing, visual attention, attachment, orientation, play, mobility, and communication. Each item is scored on a 4-point Likert scale with the following response options: “not difficult”, “slightly difficult”, “very difficult”, and “impossible”. The response option “not applicable” was treated as a missing value. In addition, this proxy-report format of the PAI-CY has a ‘parent’ component to obtain information about the issues and challenges experienced by parents (optional when the PAI-CY is used as an outcome instrument in research). Finally, a ‘sensory functioning’ component is included to give information about the sensory development of young children. The response option of “parent” and “sensory functioning” components range from “never/almost never” to “always/almost always”.

### 2.2. Main Procedures Adopted to Translation and Cultural Adaptation

To implement a validity methodology for translation and cultural adaptation, a specific approach was employed following the guidelines proposed by Beaton et al. [20]. Adhering to established frameworks, such as the back translation model outlined in the Beaton guidelines, ensures cultural relevance and contextual appropriateness, thereby reducing the risk of bias [21].

**Figure 1:**
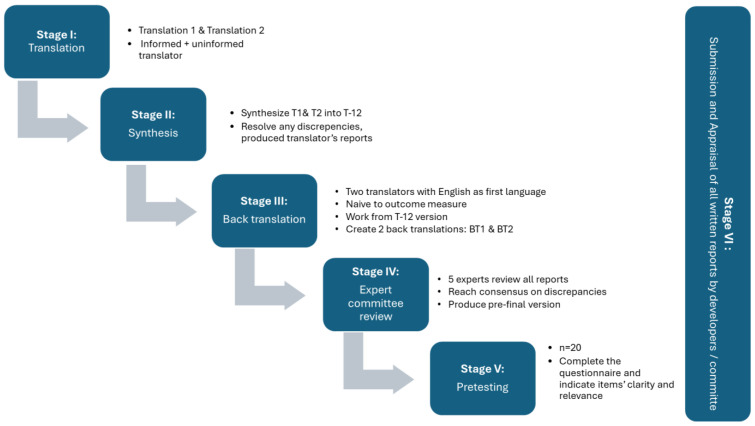
Workflow used for translation and cultural adaptation based on methodology [20].

Based on the guidelines proposed by Beaton and colleagues, the process begins with the translation and comprises a set of six stages [20], as expressed in Figure 1.

#### 2.2.1. Stage I to Stage III: Translation to Back Translation

In this first stage, two bilingual professional translators whose mother tongue is Portuguese (one familiar with developmental scales from previous work and the other unfamiliar with this type of tool) carried out two independent translations (T1 and T2). Following stage II, the two translators and the article’s 1st author had an online meeting to solve the discrepancies and obtain a consensual translated version (version T-12). A report was produced at the end of this phase, and this was documented in Zenodo. In stage III, two back translations were produced, based on version T-12. Each back translation was performed by two independent translators whose native language was English and are naïve to the tool. They produced the back translation 1 (BT1) and back translation 2 (BT2) versions, as recommended by the guidelines.

To mitigate the risk of bias, official translators and back translators from the Translators Portuguese Association database were recruited.

Back translation is a fundamental stage in the process of cultural adaptation of instruments [20,22]. Beaton and colleagues point out that it makes it possible to check whether the translation maintains the conceptual and semantic equivalence of the original version, thus guaranteeing the fidelity of the translation [20]. In addition, back translation reduces the risk of linguistic bias [22].

#### 2.2.2. Stage IV: Expert Committee Review

At stage IV an expert committee was built according to the recommendations of the authors of [20] and with the characteristics presented in Table 1. Given the need to meet the requirements expressed in the methodologies, the experts were selected on the basis of convenience. To further minimize bias, participants with diverse profiles, areas of expertise, and years of practice were included, ensuring that the main user profiles of PAI-CY were adequately represented. The committee is responsible for carrying out cross-cultural equivalence (semantic, idiomatic, experiential, and conceptual equivalence) to obtain the pre-final version of the instrument.

In the present translation and cultural adaptation, the expert panel was composed of 5 participants, according to the characteristics presented in Table 1.

First, the opinion of each expert was collected individually, regarding the semantic, idiomatic, experiential, and conceptual equivalence. For that, all the previous documentation was provided and an additional questionnaire directed to version T-12 was filled out via a digital box. That questionnaire collected information about inventory instructions, each item of each domain, and Likert scale of classifiers.

This was followed by a cognitive debriefing interview, implemented in an online system with the expert panel, to make the necessary adjustments. After the debriefing cognitive interview, and due to the lack of specific guidelines in the literature for harmonizing and reconciling an instrument, the pre-final version was again sent individually to the experts with the aim of strengthening consensus [22,23,24]. At this point, they were asked to indicate whether they agreed with the pre-final version of the instrument. A dichotomous scale (agree/disagree) was used, created by the researchers based on the instructions of the instrument, all the items that make it up, and the response format. A comment field was also created for the experts to use if necessary.

#### 2.2.3. Stage V: Pretesting

At the penultimate stage, pretesting was carried out with the target population in order to test the translation and cultural adaptation of the instrument [20].

The pre-final version was used and disseminated nationally using the digital contacts of all institutions that provide therapeutic care for infants with a visual impairment. The institutions were asked to forward the request for participation to the parents/caregivers. In this way, the sample was built randomly, and the inclusion criteria were being a parent or caregiver of a child aged between 0 and 35 months with a visual impairment and having Portuguese as a mother tongue.

In the pre-final version of PAI-CY, extra questions were added about clarity (a scale between 1—“not at all clear” to 4—“very clear” was adopted) and relevance (a scale between 1—“not all relevant” to 4—“very relevant” was adopted) [25]. In each domain a comment/suggestion box was included as well. At this stage, a socio-demographic questionnaire was implemented, inspired by previous research with PAI-CY and under the advice of the original author of the instrument [10]. The goal of this socio-demographic questionnaire was to characterize the sample that partake in the pretesting (stage V). The questionnaire consists of two sections: information about the person filling in the questionnaire (age, degree, region of residence, etc.) and information about the infant (age, sex, level of visual acuity, visual impairment, etc.). The original version can be found on Zenodo.

In the pretesting stage, the risk of bias control was also a concern. Bias was minimized through the strategy of sharing the invitation to participate across the entire country (including Portuguese islands) and among different type of institutions that support infants with a visual impairment. In addition, the data collected through the socio-demographic questionnaire allowed for the characterization of the sample and explored if any variable could influence the results.

The pretesting and socio-demographic questionnaires were available to fill out for six months, and 20 parents/caregivers from all over the country participated. The main socio-demographic characteristics of the pretesting sample are presented in Table 2.

#### 2.2.4. Stage VI: Presentation of Information to Tool Authors

At stage VI, all the documentation was detailed and presented to the contact author of the original tool in an online meeting in February 2024.

### 2.3. Statistical Analysis Used in Stage IV and Stage V

The consensus of the pre-final version of the experts was analyzed in stage IV, based on the instrument created by the researchers. For this purpose, the percentage of agree/disagree of the total number of items analyzed (including the instructions, the score scale, and items of the instrument) was calculated, considering the number of experts.

In stage V, descriptive statistics related to the main socio-demographic and clinical variable were calculated using the statistical package for the social sciences-version 29. The clarity and relevance of the PAI-CY index comprised a total of 15 items, assessed by the 20 participants, and the calculation was performed using Excel.

The main files generated through the pretesting as well as the socio-demographic questionnaire used are available on Zenodo under https://doi.org/10.5281/zenodo.10948641 (accessed on 2 January 2025).

## 3. Results

In the results section the information is organized following the workflow presented in Figure 1.

### 3.1. Stage I to Stage III: Translation to Back Translation Results

The translation and back translation process occurred according to the methodological guidelines and no major concerns were found. The completed reports of each phase can be found on Zenodo.

In total, 27 discrepancies were found between the two independent translators, mainly related to semantic and conceptual equivalence. The discrepancies and the solution implemented can be found in the Zenodo repository, and Table 3 shows examples of semantic and conceptual equivalence.

The discrepancies were solved in a meeting with the translators and the first author, and an agreement version T-12 was reached.

Following that, 54 differences were found between the two back translations and were not considered significant. A meeting was held between the two back translators and the first author, and a report was made explaining the reasons for different options. In Table 4, two examples of the aforementioned situations related to back translation are presented.

### 3.2. Stage IV: Expert Committee Results

The expert panel consisted of five participants, with their socio-demographic characteristics already described in Section 2.2.2. In the first step, the expert panel analyzed the proposed translation (sent digitally), and they marked 26 aspects as “disagree”, as shown in Table 5.

After obtaining the answers to the questionnaire evaluation by the expert committee, an online cognitive debriefing interview was conducted. During this interview, some possible changes were analyzed with the experts. Somes examples of changes introduced with this procedure were related to the elimination of the “proxy-report” expression on the cover page because there a term in Portuguese does not exist that represents the same meaning and also because usually in self-reporting measures that kind of expression is not included. Other changes were related to the clarity of the information, as in item 2 from the sensory functioning component where the expression “olhando para a luz diretamente” was replaced by “olhando diretamente para a fonte de luz”.

A digital version of the new pre-final questionnaire was sent to the experts for confirmation of the percentage of agreement.

**Figure 2:**
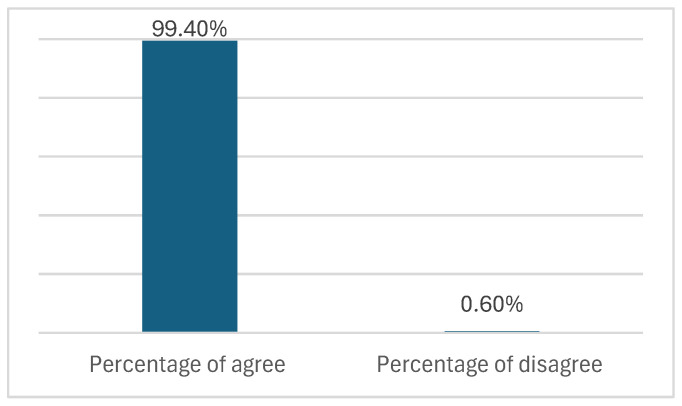
Percentage of agreement with the translation proposed, obtained in the last round of stage IV (expert committee).

Taking into account the number of items analyzed (67) and the participation of 5 experts, the total number of observations was 335. The experts agreed with the proposed translation for 333 observations, resulting in a rate of 99.40% agreement of the translated items, as shown in Figure 2.

Items 1 (which integrate stimulus processing) and 5 (which belong to visual attention) are the ones where one of the five experts did not agree with the proposed translation, which meant that 80% of the experts agreed on both items, which is why the research team kept the translation.

In this last round of the experts committee, some experts had spontaneously sent a general appreciation of the tool, such as the one transcribed below:

“*I think it’s interesting because it covers several areas. It is easy to understand and easy for parents to answer and provides important information that can guide the choice of more detailed tests and be the basis for intervention and guidance.*” Expert 2 (L)

### 3.3. Stage V: Pretesting Results

The pretesting answers were obtained over six months, with the sample consisting of 20 participants all from continental Portugal.

In Table 3, the main socio-demographic and clinical characteristics from the pretesting participants are presented.

The sample described evaluates the European Portuguese pilot version of PAI-CY in terms of clarity and relevance. As can be seen in Table 6, the responses indicated a very positive result regarding the clarity of the instructions and items. The results related to relevance were also positive, considering that a large majority of the participants rated the items and instructions as “relevant” (scored with a 3) or “very relevant” (scored with a 4). However, in 7 of the 15 items, 1 of the 20 participants indicated that the instruction was “not at all relevant” (scored with a 1) or “not very relevant” (scored with a 2). Also, note that missing values for orientation domain were detected.

These data allowed for the calculation of a scale content validity index, as proposed in the literature [26,27]. The results are the ones presented below:

$$Clarity\;PAI-CY\;Validity\;Index\;\left(C\;PAI-CY\;VI\right)=\frac{Total\;items\;scored\;with\;3\;or\;4\;}{Total\;items\;scored\;regarding\;clarity}=\frac{298}{299}≅1$$

$$Relevance\;PAI-CY\;Validity\;Index\;\left(R\;PAI-CY\;VI\right)=\frac{Total\;items\;scored\;with\;3\;or\;4\;}{Total\;items\;scored\;regarding\;relevance}=\frac{292}{299}≅0.98$$

$$Global\;PAI-CY\;Validity\;Index=\frac{C\;PAI-CY\;VI+R\;PAI-CY\;VI}{2}=\frac{1+0.98}{2}≅0.99$$

### 3.4. Stage VI: Submission and Appraisal of All Written Reports

All the translations and cultural adaptations were presented in an online meeting with the tool’s contact author. Each stage implementation was debated and approved. Additionally, strategies for future work on validation were discussed.

## 4. Discussion

The present translation and cultural adaptation were conducted following a standardized methodology, as previously described by the authors of [1]. This approach not only aligns with the recommendations of the original instrument’s authors but also ensures a systematic and rigorous process, consistent with the principles of the scientific method, representing one of the strengths of the current study.

The decision to translate and culturally adapt the English version of the Participation and Activity Inventory for Children and Youth (PAI-CY) was based on several considerations. Firstly, it was not feasible to identify four official translators with expertise in this type of process who were proficient in both Dutch and Portuguese, which would be required for a direct Dutch–Portuguese translation and back translation. Moreover, the authors of the original instrument had approved the possibility of conducting the process from the English version, which had already been used in a prior translation and cultural adaptation effort [27].

The sample obtained at each stage allows the results to be analyzed with confidence. The expert committee sample is made up of an odd number of participants, including a pediatric occupational therapist, a linguist/methodologist, and a parent. In this way, it was possible to obtain different perspectives on the translation/back translation process and improve the pretest version, as previously documented.

In addition, a very robust sample of 20 participants was obtained in the pretest phase. The pretesting of the original version included six participants [19], which allows the researchers to consider the present results with confidence. In addition, the fact that the pretest sample presents a considerable range in terms of academic degree, age, country, region, and financial situation for those who filled in the questionnaire, leads us to believe that this sample represents the diversity of Portuguese parents’ reality, minimizing the risk of bias caused by cultural or economic status, which is in line with previous literature [28].

The 99.40% approval rate set by the expert committee and the spontaneous feedback received meant that the translation and cultural adaptation could be carried out with confidence.

In the pretesting phase, the preliminary results for clarity and relevance of the PAI-CY validity indices were 1 and 0.98, respectively. In addition, the global validity index of the PAI-CY was 0.99, which was considered excellent [25,29]. Those positive results are one of the study’s advantages and grant the possibility to continue the study with the psychometric characteristics study in order to implement the validation phase (Supplementary Materials).

In addition, the coverage of the main regions of the country, as well as the different academic degree levels that exist and the financial situation of those who filled in the questionnaire, led to an adequate representation of different profiles, and is close to the sample characteristics in the original psychometric tool study [10].

As well, regarding the profiles of the infants, it was interesting to find a considerable dispersion of age (from 7 to 33 months old), covering the majority of age ranges proposed by the PAI-CY authors and close to the ones presented in the psychometric properties study of the original version [10]. The reason why infants between 25 and 33 months of age were included is because the inventory manual recommends the use of mental age, and also because the next inventory level only begins at 3 years (36 months), which means that up to 35 months of age should be included in the PAI-CY version. In addition, this option has also been analyzed and authorized by the authoring tool.

Regarding the clinical characteristics of the infants, it was interesting to understand that 45% of the situations causing visual impairment were unknown and 30% were related to cerebral damage. Previous research shows that the cerebral lesion was often the reason for visual impairment [30,31]; however, “unknown cause” was not as common as was found in the present sample [32]. Those preliminary findings about the causes of pediatric visual impairment in the Portuguese population could lead researchers to consider the necessity to implement a deeper study regarding that specific topic. Recent evidence suggests that the assessment of at-risk children may be useful to improve the early detection of children with cerebral visual impairment related vision problems [33].

The current translation and cultural adaptation into European Portuguese open up the possibility of the questionnaire being easily culturally adapted into Brazilian Portuguese, as has already been undertaken in similar translation processes [34,35]. The literature supports the use of questionnaires for pediatric visual impairment, such as cerebral visual impairment, as they can help to elicit the signs and symptoms of cerebral visual impairment and may supplement the general medical history [36].

However, some limitations identified include the fact there were no participants from the Portuguese islands (Madeira and the Azores), no caregivers filled out the questionnaire, and the clinical data were reported by parents, leading to the possibility that the questionnaire contains imprecise clinical data. The plan is to prepare the psychometric validation in order to eliminate some of these limitations, namely the lack of integrated caregivers in the sample and there being no participants from Madeira and the Azores.

## 5. Conclusions and Future Work

The present study resulted in a translated and culturally adapted version of the Participation and Activity Inventory for Children and Youth (PAI-CY) for the 0–2 years age group in European Portuguese. Despite the limitations identified in the discussion section, the translation and cultural adaptation process demonstrate very good indices. This represents an important first step toward making a valid and reliable assessment tool available for infants with a visual impairment. It also constitutes a relevant contribution to addressing an existing gap in the Portuguese clinical context and enhancing early diagnosis and intervention practices.

Future work will focus on the procedures to implement the inventory psychometric study, with the goal of producing a final, fully validated Portuguese version of the PAI-CY. In addition, data collection will be expanded beyond the scope of the pretest phase, and strategies will be implemented to address the limitations identified in the current study.

## Figures and Tables

**Table 1:** Main characteristics of expert committee participants of stage IV.

Participant	Function/Expertise Area	Years of Practice	Academic Level
A.F.	Linguist; Pediatric Speech Therapist	15	Master’s degree
J.A.	Father of a 2-year-old CVI girl	Not applicable	Master’s degree
L.N.	Senior Pediatric Occupational Therapist	38	Bachelor’s degree
R.A.	Pediatric Occupational Therapist specialized in visual impairment	12	Master’s degree
R.S.	Lecturer in Occupational Therapy degree course	18	Ph.D. degree

**Table 2:** Socio-demographic and main clinical characteristics of participants (n = 20).

Socio-Demographic Characteristics		Clinical Characteristics	
Family member who completed the questionnaire, n (%)		Infant age (in months), mean ± SD (range)	22.50 ± 7.07 (7–33)
Mother	16 (80%)		
Father	2 (10%)	Male gender n (%)	11 (55%)
Both	2 (10%)		
		Infant during the day	
Academic degree of who filled out n (%)		Daycare	11 (55%)
1st cycle	1 (5%)	Nanny	2 (10%)
2nd cycle	0 (0%)	Other	7 (35%)
3rd cycle	2 (10%)		
Secondary education	7 (35%)	Infant acuity n (%)	
Bachelor	4 (20%)	Normal	4 (20%)
Master	6 (30%)	Low vision	12 (60%)
Age of who filled out the questionnaire (in years), mean ± SD (range)	34.16 ± 5.82 (22–43)	Other condition	4 (20%)
Country region n (%)		Cause of visual deficit n (%)	
North	1 (5%)	Cerebral damage	6 (30%)
Center	10 (50%)	Retina damage	2 (10%)
Lisbon metropolitan area	8 (40%)	Optical damage	2 (10%)
Alentejo	1 (5%)	Unknown	9 (45%)
Financial situation parent, n (%)		Developmental pathology: yes n (%)	12 (60%)
Good income	6 (30%)		
Generally sufficient income	6 (30%)		
Sufficient income	5 (25%)		
Insufficient income	2 (10%)		
Don’t know/no answer	1 (5%)		

**Table 3:** Examples of semantic and conceptual equivalence obtained in stage II (Synthesis).

Original Text (English)	Version T1	Version T2	Version T 12 (Final Agree Version/Synthesis)	Justification/ Comments
Parent(s)/*caretaker(s)*	Pais/*responsáveis*	Pais/*encarregados de educação*	Pais/cuidadores	Conceptual Equivalence: the word “cuidadores” expresses more precisely the diversity of people taking care of a child.
Even when *you find* a question unimportant or do not know the answer, we still *ask* you to select the most suitable answer. *When in doubt about the answer*, choose the answer that best fits the situation.	Mesmo *quando considera* que uma pergunta não é importante *ou não sabe* a resposta, *pedimos que selecione a* resposta mais adequada. Em caso de dúvida sobre a resposta, assinale a que melhor se adequa à situação.	Mesmo *considerando* que uma pergunta não é importante ou *que* não sabe a resposta, *sugerimos que opte pela* resposta mais adequada. Caso *tenha dúvidas acerca de alguma resposta*, assinale a que melhor se adequa à situação.	Mesmo quando considera que uma pergunta não é importante ou que não sabe a resposta, pedimos que opte pela resposta mais adequada. Caso tenha dúvidas acerca de alguma resposta, assinale a que melhor se adequa à situação.	Semantic Equivalence: more coherent, considering the original text.

Note: The words or expressions with discrepancies are written in italic.

**Table 4:** Examples of differences detected between the two back translation and the explanation finding in an online meeting.

Page	Original Item	Version BT1	Version BT2	Back Translators’ Analyses
1	Aims to identify	*Intends* to identify	Aims *at identifying*	Consistent translation—synonyms
2	1. React to visual stimuli (for example, does your child react by laughing, crying or extending their arms if you hold a toy in front of him/her)?	1. React to visual stimuli (for example, does your child react by laughing, crying or extending their arms if you hold a toy in front of *them*)?	1. React to visual stimuli (for example, does your child *laugh, cry or stretch out his/her arms when* you hold a toy in front of him/her)?	Consistent translation-Plural/singular referring to “children” instead of “child”. Synonyms Syntactic equivalence

Note: The words or expressions with discrepancies are written in italic.

**Table 5:** Expert panel evaluation.

	Items in Disagreement
E 1 (A)	Instructions—disagree Activity and participation component—disagree Stimulus processing—item 1—disagree Visual attention—item 5—disagree Bonding—item 9—disagree Communication—items 25 and 26—disagree Parent instructions—disagree Parent component—item 1—disagree Sensory functioning component—items 2, 3 and 4—disagree
E 2 (L)	Instructions—disagree Activity and participation component—disagree Play—item 21—disagree Parent instructions—disagree Parental component—item 7—disagree
E 3 (J)	Stimulus processing—item 1—disagree Play—item 23—disagree Sensory functioning component—item 2—disagree
E 4 (RA)	Visual attention—item 5—disagree Mobility—item 17—disagree Other—namely—disagree
E 5 (R)	Visual attention—item 5—disagree Mobility—item 17—disagree Other—namely—disagree

Abbreviations: E—expert.

**Table 6:** Result from clarity and relevance of IPA CY instructions and domains (n = 20).

	Clarity Assessment in Pretesting Phase				Relevance Assessment in Pretesting Phase			
	Not at All Clear (1)	Unclear (2)	Clear (3)	Very Clear (4)	Not at All Relevant (1)	Not Very Relevant (2)	Relevant (3)	Very Relevant (4)
General instruction		1	5	14		1	4	15
Activity and participation component instruction			6	14			7	13
Stimulus processing			3	17		1	8	11
Visual attention			4	16			5	15
Bonding			3	17		1	5	14
Orientation			4	15	1		6	12
Mobility			3	17	1		4	15
Play			3	17		1	4	15
Communication			3	17			4	16
Parent component instruction			3	17			4	16
Parent component			3	17			4	16
Sensory functioning component instruction			3	17			4	16
Sensory functioning			5	15			4	16
Visual aids			3	17		1	4	15
Overall mood			3	17			4	16
Total obtained:	0	1	54	244	2	5	71	221
Total in agreement (score 3 or 4)			298				292	
Total in disagreement (score 1 or 2)	1				7			

Note: Missing values were detected in orientation domain (both for clarity and relevance).

## Data Availability

The data generated in the present research are available and open at the scientific repository Zenodo under the doi: 10.5281/zenodo.10948641.

## Supplementary Materials

The following supporting information can be downloaded at: https://www.mdpi.com/article/10.3390/brainsci15040394/s1, File S1: PAI_CY translated and culturally adapted for European Portuguese.

## Abbreviations

The following abbreviations are used in this manuscript:

BT1	Back translation 1
BT2	Back translation 2
C PAI-CY VI	Clarity PAI-CY Validity Index
PAI-CY	Participation and Activity Inventory for Children and Youth
R PAI-CY VI	Relevance PAI-CY Validity Index
WHO	World Health Organization
