# Supplementary material for: Participation and Activity Inventory for Children and Youth (PAI-CY): Translation and Cultural Adaptation to European Portuguese
Source: Brain Sci. 2025 Apr 13;15(4):394. doi: 10.3390/brainsci15040394 (PMC12025917; doi:10.3390/brainsci15040394)
Supplement: Supplementary file 1 [file brainsci-15-00394-s001.zip › brainsci-3530928-supplementary.pdf]

Inventário de Participação e Atividade para Crianças e Jovens
- Dois primeiros anos de idade -

Elsman, R., Nispen, R. & Rens, G. (2019)
com tradução a adaptação transcultural de Ferreira, A. I., Santana, M. R., Quaresma, C. & Quintão, C.

Nome da criança: ____________________________ DN: ___/ ___/___ Idade: _______

Inventário preenchido por: _____________________________________ Data: __/__/__

Instruções

Este questionário permite identificar as dificuldades percecionadas pelos pais / cuidadores dos bebés com défice visual e idades entre os 0 e os 35 meses.

Ao preencher o questionário selecione a opção de resposta mais adequada para cada pergunta. Não pense demasiado, pois geralmente a primeira resposta que vem à mente é a melhor. Mesmo quando considera que uma pergunta não é importante ou que não sabe a resposta, pedimos que selecione a opção que considera mais adequada. Caso tenha dúvidas acerca de alguma resposta, assinale a que melhor se adequa à situação.

O questionário é constituído por 3 componentes: uma componente de atividade e participação, uma componente parental e uma componente de funcionamento sensorial. No final também são feitas perguntas sobre dispositivos de apoio visual e o humor habitual do bebé.

O questionário destina-se a bebés dos 0 aos 2 anos e o seu preenchimento demora cerca de 10 minutos. Agradecemos o preenchimento do mesmo.

O presente documento é o resultado do trabalho desenvolvido no pré-teste do instrumento
"Inventário de Participação e Atividade para Crianças e Jovens - dois primeiros anos de idade",
para português europeu.

Componente de atividade e participação

As perguntas que se seguem referem-se às atividades que o seu bebé realiza diariamente. O objetivo deste questionário é obter uma melhor perceção das dificuldades que o bebé apresenta ao realizar as atividades. As dificuldades podem ser causadas pelo défice visual, por problemas de processamento visual e também por outros problemas ou pelas características do temperamento do bebé.

Ao responder às perguntas, assinale a opção de resposta que melhor se aplica. Desta forma, não é relevante se as dificuldades advêm de problemas de visão/de processamento visual ou de quaisquer outros problemas. A opção de resposta "N/A" (não aplicável) só deve ser assinalada caso a atividade não seja pertinente para o bebé, por exemplo, se não está a realizar uma atividade por outros motivos. Se o seu bebé usa dispositivos de apoio visual (como óculos ou ampliador), responda às perguntas assumindo que estão a ser utilizados esses recursos.

No final do questionário, pode indicar que dispositivos de apoio visual o bebé utiliza.

Processamento de estímulos – Que grau de dificuldade apresenta o seu bebé para:

	Sem dificuldade	Um pouco de dificuldade	Dificuldade moderada	Muita dificuldade	Impossível	N/A
1. Reagir a estímulos visuais (por exemplo, o bebé ri, chora ou estende os braços na sua direção quando você segura um brinquedo à sua frente)?	○	○	○	○	○	○
2. Reconhecer sons familiares (por exemplo, a campainha, uma voz, a máquina de lavar)?	○	○	○	○	○	○
3. Reagir a gestos inesperados (por exemplo, ser pegado ao colo)?	○	○	○	○	○	○

Atenção visual – Que grau de dificuldade apresenta o seu bebé para:

	Sem dificuldade	Um pouco de dificuldade	Dificuldade moderada	Muita dificuldade	Impossível	N/A
4. Observar especificamente algo que está longe (por exemplo, um avião ou um pássaro).	○	○	○	○	○	○
5. Olhar para algo durante um longo período (por exemplo, fotografias)?	○	○	○	○	○	○
6. Alternar a atenção visual (por exemplo, olhar primeiro para a mãe e depois para o pai)?	○	○	○	○	○	○

Relacionamento de proximidade – Que grau de dificuldade apresenta o seu bebé para:

	Sem dificuldade	Um pouco de dificuldade	Dificuldade moderada	Muita dificuldade	Impossível	N/A
7. Reconhecer expressões faciais?	○	○	○	○	○	○
8. Imitar expressões faciais?	○	○	○	○	○	○
9. Reconhecer o rosto de pessoas familiares até um máximo de aproximadamente 4 metros de distância (por exemplo, pais e familiares)?	○	○	○	○	○	○
10. Imitar ações (por exemplo, bater palmas)?	○	○	○	○	○	○
11. Imitar sons?	○	○	○	○	○	○
12. Explorar uma divisão da casa de forma autónoma?	○	○	○	○	○	○

Orientação – Que grau de dificuldade apresenta o seu bebé para:

	Sem dificuldade	Um pouco de dificuldade	Dificuldade moderada	Muita dificuldade	Impossível	N/A
13. Orientar-se numa divisão da casa (por exemplo: o seu bebé sabe onde encontrá-lo(a) se ela estiver a brincar noutra divisão)?	○	○	○	○	○	○
14. Explorar o espaço envolvente, utilizando o toque?	○	○	○	○	○	○

Mobilidade – Que grau de dificuldade apresenta o seu bebé para:

	Sem dificuldade	Um pouco de dificuldade	Dificuldade moderada	Muita dificuldade	Impossível	N/A
15. Levantar a cabeça?	○	○	○	○	○	○
16. Rolar?	○	○	○	○	○	○
17. Gatinhar/rastejar de barriga para baixo/rastejar de rabo?	○	○	○	○	○	○
18. Sentar-se?	○	○	○	○	○	○
19. Pôr-se de pé?	○	○	○	○	○	○
20. Manter-se de pé de modo autónomo?	○	○	○	○	○	○
21. Caminhar com apoio?	○	○	○	○	○	○

Brincadeira – Que grau de dificuldade apresenta o seu bebé para:

	Sem dificuldade	Um pouco de dificuldade	Dificuldade moderada	Muita dificuldade	Impossível	N/A
22. Ver fotos/imagens juntos?	O	O	O	O	O	O
23. Ler livros juntos?	O	O	O	O	O	O
24. Manipular brinquedos/jogos (por exemplo, carregar nos botões dos brinquedos/jogos)?	O	O	O	O	O	O
25. Brincar com outros bebés e/ou crianças?	O	O	O	O	O	O

Comunicação – Que grau de dificuldade apresenta o seu bebé para:

	Sem dificuldade	Um pouco de dificuldade	Dificuldade moderada	Muita dificuldade	Impossível	N/A
26. Compreender gestos simples (com mãos) (por exemplo, apontar ou acenar)?	O	O	O	O	O	O
27. Compreender a linguagem (por exemplo, o bebé move-se na sua direção quando o chama)?	O	O	O	O	O	O

Componente parental

As perguntas que se seguem referem-se às suas experiências como pai/mãe/cuidador. Na questão 4, a opção de resposta "não aplicável" só deve ser assinalada se não tiver um(a) companheiro(a) e na questão 7, se o bebé não tiver irmãos(ãs).

	Nunca/ quase nunca	Às vezes	Regularmente	Frequentemente	Sempre/ quase sempre	N/A
1. Com que frequência realiza tarefas difíceis que competem ao seu bebé?	○	○	○	○	○	○
2. Com que frequência compartilha com outros o cuidado do seu bebé (família, amigos, ama)?	○	○	○	○	○	○
3. Com que frequência recebe apoio de amigos ou familiares?	○	○	○	○	○	○
4. Com que frequência recebe apoio do(a) seu(sua) companheiro(a)?	○	○	○	○	○	○
5. Com que frequência se preocupa com o futuro do seu bebé?	○	○	○	○	○	○
6. Com que frequência sente angústia pelo facto do seu bebé ser "diferente"?	○	○	○	○	○	○
7. Com que frequência consegue dividir a sua atenção pelas/os crianças/bebés ao seu cuidado?	○	○	○	○	○	○

Componente do funcionamento sensorial

As perguntas que se seguem têm como objetivo obter uma visão geral do funcionamento sensorial do bebé.

	Nunca/ quase nunca	Às vezes	Regularmente	Frequentemente	Sempre/ quase sempre	N/A
1. Com que frequência o bebé reage à luz, desviando o olhar ou fechando os olhos?	O	O	O	O	O	O
2. Com que frequência o bebé reage à luz, olhando diretamente para a fonte de luz?	O	O	O	O	O	O
3. Com que frequência o bebé reage ao som, procurando-o?	O	O	O	O	O	O
4. Com que frequência o bebé explora objetos com a boca?	O	O	O	O	O	O
5. Com que frequência o bebé prefere brinquedos com som?	O	O	O	O	O	O
6. Com que frequência o seu bebé prefere brinquedos com cores contrastantes (por exemplo, vermelho/amarelo)?	O	O	O	O	O	O
7. Com que frequência o seu bebé embate contra coisas?	O	O	O	O	O	O
8. Com que frequência o bebé vira a cabeça quando tenta estabelecer contacto com ela?	O	O	O	O	O	O

Que dispositivos de apoio visual o seu bebé utiliza?

	Sim	Não	Não sei
Óculos/lentes de contacto	O	O	O

Outros, especifique.

De um modo geral, como descreve o humor do seu bebé?

- ○ Assustado
- ○ Zangado
- ○ Feliz
- ○ Triste
- ○ Neutro
- ○ Outros, especifique.

Observações:

O presente documento é o resultado do trabalho desenvolvido no pré-teste do instrumento "Inventário de Participação e Atividade para Crianças e Jovens - dois primeiros anos de idade", para português europeu.
